# Alternative Treatment Method for Novel Coronavirus Disease 2019: Coupled Plasma Filtration Adsorption

**DOI:** 10.5152/eurasianjmed.2021.20151

**Published:** 2021-06

**Authors:** Bahadir Ciftci, Cem Erdogan, Deniz Kizilaslan, Mursel Ekinci, Oktay Olmuscelik, Yunus Oktay Atalay

**Affiliations:** 1 Department of Anesthesiology and Reanimation, İstanbul Medipol University, School of Medicine, İstanbul, Turkey; 2 Department of Internal Medicine, İstanbul Medipol University, School of Medicine, İstanbul, Turkey

Dear editor:

Patients infected with severe acute respiratory syndrome corona virus 2 may have mild symptoms such as fever and cough in the early stage of this disease and these symptoms may deteriorate to acute respiratory distress syndrome, multiple-organ failure, and death in the late phase.^1^ It has been reported that the cytokine levels increase and damage the tissues.^2^ As some agents used to decrease cytokine levels such as intravenous immunoglobulin and selective cytokine blockade (eg, anakinra or tocilizumab) may cause adverse events (infections, allergic reactions, and changes in blood pressure), there is a need of alternative treatment methods.^3^ Herein, we present our coupled plasma filtration adsorption (CPFA) (a detoxification system combining a plasma adsorption circuit) with a continuous renal replacement therapy (CRRT) experiences to treat 2 patients with coronavirus disease (COVID-19) in our tertiary university hospital intensive care unit (ICU).

Written consent was obtained from the patients. The first patient was a 54-year-old man and the second was a 55-year-old woman. Both the patients were diagnosed with COVID-19 by specific molecular test (PCR) and thorax tomography. The man had a comorbidity (hypothyroidism). Both patients had tachypnea and dyspnea at the time of ICU admission (respiratory rate 40/min and 43/min, respectively). They had fever (38.5 °C and 39.2 °C, respectively). IL-6, D-Dimer, and Ferritin levels of the patients are shown in Table 1. As a part of the protocol, IL-6 levels are routinely measured for every patient with COVID-19 in our clinic. Their saturations levels were 75% and 70% with 10 L/min oxygen flow, respectively. After supporting their respiration with noninvasive mechanical ventilation, we had to intubate them. Hemodynamic parameters of the patients were stable, there was no need to the inotropic agents. The creatinine levels of the patients were in normal range. The clinical status and cytokine levels were unresponsive to our COVID-19 treatment protocol (chloroquine, favipiravir, and tocilizumab). Then, we decided to start CPFA on Amplya (Bellco, Medtronic, US) using a membrane (Medisorb, Bellco, Medtronic, US) to remove the cytokines from the blood.

The cytokine levels of the patients decreased significantly. At day 5 and day 6 of CPFA, respectively, they were extubated. Two consecutive PCR tests of the patients were negative. They recovered from COVID-19 completely and were discharged to clinic within 15 days. In several studies, it has been reported that the cells secrete high levels of proinflammatory cytokines (IL-1β, IL-6, and tumor necrosis factor [TNF]) in patients with COVID-19.^2^,^3^ Unfortunately, there is no proven effective therapy to decrease the cytokine levels, and the clinical trials to find one have been going on.^1^–^3^ As reported in septic shock-associated acute renal failure, CRRT with sepsis membrane effectively removes TNF-α, IL-6, IL-8, and IFN γ.^4^ Therefore, the following question arises: would CRRT decrease cytokine levels in patients with COVID-19? The removal of pathogenic blood constituents from the plasma may also prove to be beneficial for these patients. In some studies, it has also been reported that therapeutic plasma exchange may be used successfully as a first step therapy.^5^ Therefore, we thought that a combination of plasma adsorption circuit with CRRT may be beneficial to our patients. The cytokine levels were not as high to cause a cytokine storm. First, we used tocilizumab. However, there was no response, and the cytokine levels did not decrease. Then, we used CPFA, and improvement was observed in the clinical status and cytokine levels. Therefore, it can be concluded that CPFA may be an option to treat COVID-19 infection.

## Figures and Tables

**Table 1:** The Laboratory Levels of the Patients. The Yellow Areas of the Tables Are the Application Period of CPFA

PATIENT 1	1.DAY	2.DAY	3.DAY	4.DAY	5.DAY	6.DAY	7.DAY	8.DAY
IL-6	x	x	396,2	x	88,13	37,85	27,27	17,79
FERRITIN	3702,00	4078	3598,5	x	2567,5	1778	955	1061
D-DIMER	7911,91	x	x	x	2766,69	x	x	3675,21
PATIENT 2								
IL-6	x	78,41	x	x	68,11	x	18,11	x
FERRITIN	4100	3450	x	x	x	345,8	95,5	x
D-DIMER	x	x	x	x	x	x	x	x
